# Effect of Sheng-Jiang Powder on Obesity-Induced Multiple Organ Injuries in Rats

**DOI:** 10.1155/2017/6575276

**Published:** 2017-10-29

**Authors:** Juan Li, Yu-mei Zhang, Jun-yi Li, Lv Zhu, Hong-xin Kang, Hong-yu Ren, Huan Chen, Ling Yuan, Yi-fan Miao, Mei-hua Wan, Wen-fu Tang

**Affiliations:** Department of Integrated Traditional Chinese and Western Medicine, West China Hospital, Sichuan University, Chengdu, Sichuan Province 610041, China

## Abstract

**Background and Aims:**

Obesity has become the main public health issue nowadays with poor control and has been associated with increased risk of multiorgan disease, but the specific mechanism and effective medication are still to be addressed. Sheng-jiang powder (SJP) showed great potential in preventing obesity in Chinese researches but has no trace in English reports. This study was designed to investigate the effect of SJP on obesity and obesity-mediated multiorgan injuries.

**Methods:**

Rats were randomized into normal group (NG), obese group (OG), and SJP treatment group (SG). Obesity was induced by high-fat diet feeding. Rats were gavaged with SJP/normal saline daily from the third week and all rats were sacrificed after 12 weeks' feeding. Tissues were obtained for cytokines tests.

**Results:**

Firstly, high-fat diet feeding led to significant obesity. Compared to NG, the level of SOD in the liver, spleen, lung, and kidney was much lower in OG (*p* < 0.05), while the pathological scores of pancreas, liver, spleen, lung, and kidney were much higher. SJP significantly increased SOD level in the liver, spleen, and lung and reduced the pathological scores of pancreas, liver, spleen, lung, and kidney correspondingly (*p* < 0.05).

**Conclusions:**

SJP ameliorates inflammatory response and mitigates obesity-induced multiple organ injuries.

## 1. Introduction

The rising prevalence of obesity has achieved an unprecedented pandemic around the world. According to the results of Global Burden of Disease 2013 Study (GBD), the prevalence of overweight and obesity combined has risen by 27.5% for adults and 47.1% for children globally during the past three decades. And the number of overweight and obese individuals has increased to 2.1 billion in 2013, which is 2.28 times that of 1980 [1]. Obesity has been related to an increased risk of a series of diseases that involve multiple organ-systems of the body and was estimated to cause 3.4 million deaths, 3.9% of years of life loss, and 3.8% of disability adjusted life years (DALYs) globally in 2010 [2]. The specific mechanisms of obesity leading to diseases have not yet been fully elucidated, but increasing evidences have linked obesity to inflammation according to the work over the past decades.

Obesity-associated inflammation is a chronic, persistent, low-grade inflammation but with insidious effect against multiple organs. Lipid overaccumulation and energy metabolism disorder lead to inflammatory changes of microenvironment of adipose tissue with abundant macrophage infiltration, unbalanced secretion of adipokines, and overexpression of proinflammatory cytokines [3]. Subsequent activation of several inflammation signal pathways by those cytokines helps amplify the inflammatory response, which in turn promotes the expression and secretion of proinflammatory cytokines and eventually leads to inflammatory injuries of all organs without a single one having narrow escape [4]. Studies in recent years have demonstrated the independent role of obesity in the development of a series of diseases, such as nonalcoholic fatty liver disease [5], cardiovascular disease [6, 7], skeletal and muscular disorders [8], intestinal microbiota imbalance [9], metabolic disorders [10], respiratory disease [11], kidney disease [12], and neurological disease [13, 14], while the liver, heart, pancreas, bone, muscle, brain, and many other organs are relevant [15]. Therefore, increasing trials are carried out which try to find proper methods or drugs to control the ongoing trend of obesity and relevant organ injuries, such as sea buckthorn leaves extract [16], shao fu zhu yu decoction [17], anthocyanin-rich foods [18], and probiotics supplement [9].

According to the traditional Chinese medicine (TCM) theory, obesity belongs to the category of “Turbidity,” a syndrome caused by “ascending and descending disfunction” of spleen [19]. Spleen disfunction leads to abnormal motion of qi, further cause qi stagnation, phlegm retention, and blood stasis, and finally induces the occurrence of obesity. To treat turbidity is to regulate the generation, transportation, and distribution of lipid. And the permanent cure is to resume the “ascending and descending” function of spleen. SJP is derived from “wan bing hui chun,” which was compiled by ting-xian gong during the Ming dynasty of China, and consists of Jiangchan* (Bombyx Batryticatus)*, Chantui* (periostracum cicada)*, Jianghuang* (Curcuma longa)*, and Dahuang* (Rheum palmatum)* [20]. As a classic representative formula to treat “ascending and descending disfunction,” SJP was demonstrated to be effective in lowering body weight and anti-inflammation, antiviral, antiallergic, antipyretic, and immune regulation [21]. Early in the 1990s, there had been a study focused on the effect of SJP combined with auricular point sticking in lowering body weight [22]. SJP combined with acupuncture treatment can significantly increase serum adiponectin level, decrease serum leptin and intracellular ROS expression, and mitigate obesity-related inflammation in obese patients [23, 24]. However, almost all studies about SJP were reported in Chinese, and the effects of SJP on obesity-related multiple organ injuries have not been fully elucidated so far. Therefore, we designed this study to explore the effect of SJP on obesity-related inflammatory damage of multiple organs to give the world a comprehensive impression about SJP in ameliorating obesity-associated multiple organ injuries.

## 2. Materials and Methods

### 2.1. Design

This study is a prospective, randomized controlled trial.

### 2.2. Settings

The study was set at Ethnopharmacology Laboratory at West China Hospital.

### 2.3. Ethics Statement

The protocol was approved by the Ethics Committee for Animal Experiments of Sichuan University. All rats were handled according to the University Guidelines and the Animal Care Committee Guidelines of West China Hospital. All surgeries were performed under chloral hydrate anesthesia, and all efforts were made to minimize suffering of rats.

### 2.4. Preparation of Sheng-Jiang Powder

Sheng-jiang powder (SJP) was derived from the famous Chinese medical book “wan bing hui chun,” and was composed of Jiangchan (*Bombyx Batryticatus*, 6 g), Chantui (*periostracum cicada*, 3 g), Jianghuang (*Curcuma longa*, 9 g), and Dahuang (raw rhubarb, 12 g). Jiangchan (1701117), Chantui (1608027), Jianghuang (1506067), and Dahuang (1610039) were purchased from Chengdu New Green Herbal Pharmaceutical Co., Ltd. (Chengdu, China). The crude drugs were identified and the prescription for this study was an aliquot from the same batch. SJP was boiled twice in distilled water (1 : 12, w/v) for 30 min each time. The blended supernatants were then lyophilized (yield = 23% w/w, dried extract/crude drug). The dried extract was dissolved in distilled water before use. According to the original prescription recorded, the dose of an adult was 0.5 g/Kg·BW. Therefore, we adopt a 10-fold dose (5 g/Kg·BW) to treat the experimental animals.

### 2.5. Animals and Treatment

**Figure 1:**
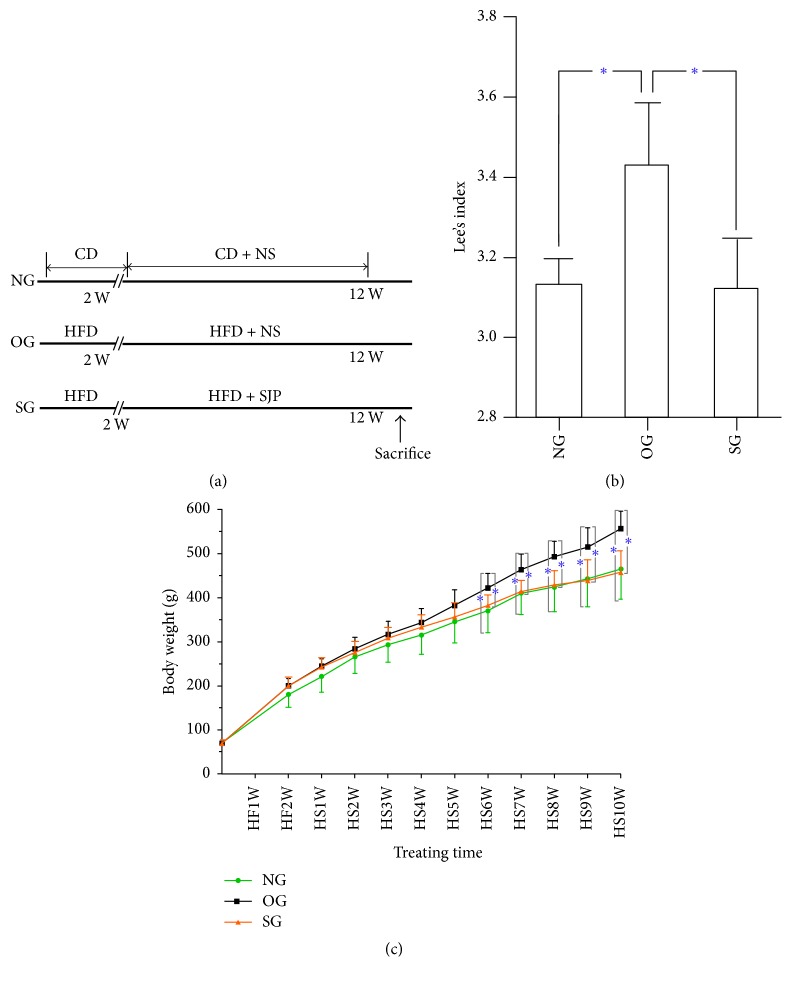
*Study design of feeding methods and the body weight and Lee's index of rats with high-fat diet feeding with/without Sheng-jiang powder (SJP) administration*. Normal group (NG), obese group (OG), and SJP treatment group (SG). CD: chew diet; HFD: high-fat diet; NS: normal saline; SJP: Sheng-jiang powder. HF: high-fat diet feeding; HS: high-fat diet feeding and SJP administration. (a) Feeding and intervention methods of the study; (b) Lee's index of rats before sacrifice; (c) body weight of rats in the three experimental groups during the whole process of feeding. The whole study lasted 12 weeks with 10 weeks' administration of SJP (5 g/Kg) one time a day. All rats were sacrificed after 12 weeks' feeding. *∗* indicates that *p* < 0.05.

Male Sprague-Dawley rats weighed 60–80 g were purchased from Chengdu Dashuo Experimental Animal Co., Ltd (Chengdu, China). All animals were kept under controlled temperature (22-23°C) and on a 12-h light/12-h dark cycle and had free access to a high-fat diet (60% of calories derived from fat; TP23300; Trophic Animal Feed High-tech Co., Ltd, China) to induce obesity or control diet (16.7% of calories derived from fat; TP23302; Trophic Animal Feed High-tech Co., Ltd, China) (http://trophic.biomart.cn). Animals were randomly allocated to normal group (NG, control diet, *n* = 6), obese group (OG, high-fat diet, *n* = 8), and Sheng-jiang powder group (SG, high-fat diet plus Sheng-jiang powder, *n* = 8) by random number table. The whole study lasted for 12 weeks with 10 weeks' administration of SJP (5 g/Kg) one time a day and body weight was recorded every week. Rats in SG were gavaged with SJP from the third week, while rats in the other two groups were gavaged with equal volume of normal saline instead. All rats were sacrificed after 12 weeks' feeding (Figure 1(a)). Tissue samples were obtained for cytokines tests and histopathological analysis. This study adhered to the ARRIVE Guidelines for reporting animal research (S1 ARRIVE Checklist).

### 2.6. Tissue Sampling and Cytokines Analysis

All rats were sacrificed after 12 weeks' feeding and blood samples were obtained from heart. Liver, heart, spleen, lung, kidney, intestine, and pancreas tissues were dissected immediately and collected for cytokines and histopathological analysis. Tissue samples were homogenized using a tissue homogenizer (Biospec Products, Bartlesville, OK). Homogenates were incubated at 4°C for 30 min and then centrifuged at 1000 ×g for 10 minutes. Supernatants were collected for cytokine analysis. Malondialdehyde (MDA), superoxide dismutase (SOD), glutathione peroxidase (GSH-px), reactive oxygen species (ROS), and myeloperoxidase (MPO) were measured by means of enzyme-linked immunosorbent assay (ELISA) (eBio, Wuhan, China) with commercially available materials. According to the manufacturer's protocol, absorbance was measured at 450 nm with High Throughput Universal Microplate Assay. The sample values were then read off the standard curve and the relative concentrations were calculated.

### 2.7. Histopathological Analysis

Fresh tissue samples were fixed in 10% neutral formalin and embedded in paraffin and then sectioned into 5 *μ*m slices and followed with hematoxylin and eosin (H&E) staining. All the histopathology specimens were reviewed and scored in a blinded fashion by two independent pathologists using a scoring system for the extent and severity of tissue injury (points 0–4, edema, neutrophil infiltration, necrosis, and hemorrhage) as previously described [25]. The total histopathology score is the mean of the combined scores for each parameter from both investigators.

## 3. Statistical Analysis

All data were expressed as mean ± SD. Statistical analysis was performed with PEMS3.1 statistical program for Windows. One-way ANOVA was used to analyze group differences in the study. Differences with a *p* < 0.05 were considered to be statistically significant.

## 4. Results

### 4.1. SJP Protect against High-Fat Diet Induced Obesity in Experimental Rats

Obesity were successfully induced after 8 weeks' high-fat diet feeding with the body weights of rats in OG significantly increased by 20% more than that of the NG. However, rats in SG showed a much slower weight gain with SJP gavage and the body weights of rats in SG were significantly lower than that of OG after 6 weeks' gavage. At the end of the experimental period, rats in NG and SG showed an almost similar body weight gain and Lee's index; both were significantly lower than that of OG (Figures 1(b) and 1(c)).

### 4.2. SJP Ameliorate Tissue Inflammation of Obese Rats

Obesity led to distinct changes of cytokines in tissue samples of rats. In our study, according to all the indicators we selected in different tissues, obesity contributed to the significant elevated level of MDA (a product of lipid peroxide degradation which reflects the degree of oxidative stress response) in heart and GSH-px (a peroxide decomposition enzyme) in liver, while it decreased level of SOD (a free radical scavenger) in liver, spleen, lung, and kidney. However, SJP inversely decreased the level of MDA in heart and GSH-px in liver and elevated the level of SOD in liver, spleen, lung, and kidney (Table 1).

### 4.3. SJP Mitigate Multiple Organ Injuries in Obese Rats

**Figure 2:**
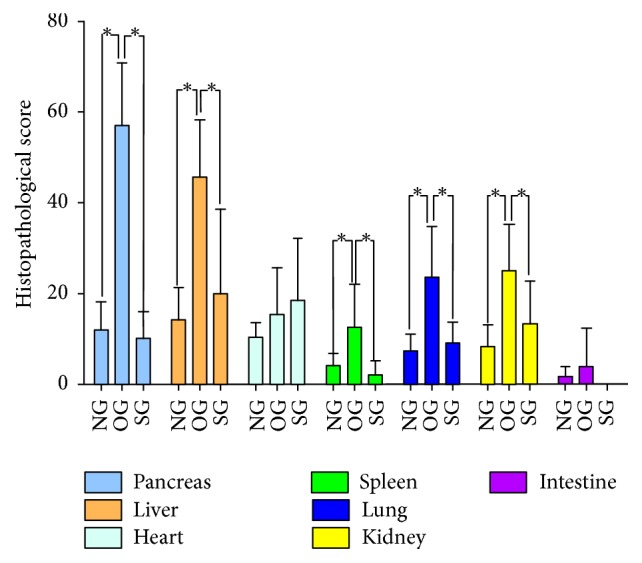
*The pathologic scores of rats' organs in all of the three experimental groups*. *∗* indicates that *p* < 0.05.

**Figure 3:**
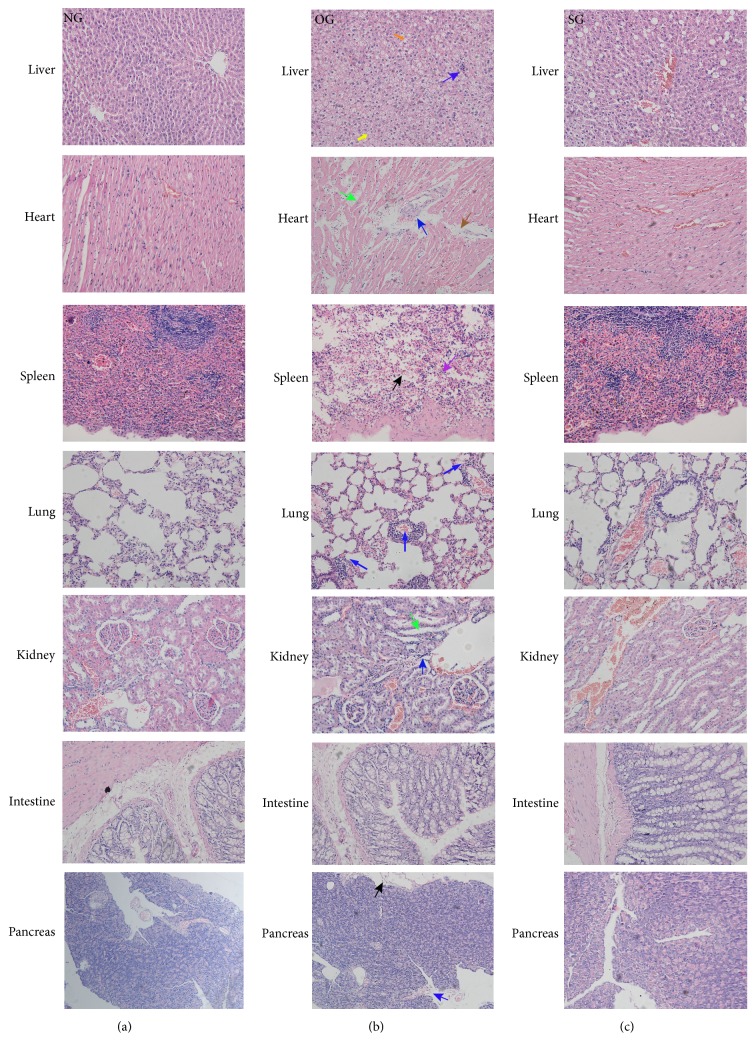
*The histological images of rats' organs in all of the three experimental groups*. Hematoxylin-eosin counterstain. Histological images are presented with original magnification 200x. The histological images of rats' organs from (a)–(c) exhibited tissue damage of rats in NG, OG, and SG, separately. Distinct changes were observed in different organs of obese rats (b), including enlarged hepatocytes (yellow arrow), extensive vacuolization (orange arrow), inflammatory cells infiltration (blue arrow), tissue edema (green arrow), myocardial early infarction (brown arrow), follicular degeneration (purple arrow), and necrosis (black arrow). These histological changes were partly reversed by SJP administration for 10 weeks (5 g/Kg·bw/day) (c).

Inflammation led to obvious tissue damage of multiple organs in obese rats. The histopathological evaluation results uncovered significant higher pathological scores of pancreas, liver, spleen, lung, and kidney of obese rats with more inflammatory cell infiltration and/or much severe tissue edema or cell vacuolation or cell necrosis. Reversely, SJP distinctly lowered the pathological score of pancreas, liver, spleen, lung, and kidney with less inflammatory cell infiltration and/or mild tissue edema or less cell vacuolation and necrosis (Figures 2 and 3).

## 5. Discussion

In the present study, we investigated the effect of SJP on systemic inflammatory injuries of multiple organs in obese rats. Our results uncovered significantly lower expression of SOD in the liver, spleen, lung, and kidney of obese rats and more severe injuries of pancreas, liver, spleen, lung, and kidney, while SJP was effective in increasing tissue levels of SOD in the liver, spleen, and lung and ameliorating inflammatory injuries of pancreas, liver, spleen, lung, and kidney correspondingly.

According to traditional Chinese Medicine theory, SJP was applied in “febrile symptoms” for evacuating wind and clearing heat, ascending lucidity, and descending turbidity. With the deepening of the studies to the mechanism of obesity and the characters of the formula, SJP was found quite appropriate to treat obesity and first reported in the treatment of Norplant subcutaneous preparations induced obesity for its prominent effect of lowering body weight [22, 24]. Studies afterwards further demonstrated that SJP was effective in anti-inflammation and immune regulation and was widely used in inflammatory diseases, such as flu, asthma, glomerulonephritis, acute pancreatitis, sepsis, and obesity.

Obesity-induced inflammation is a chronic, low-grade inflammation first started in adipose tissue with abundant macrophage infiltration and then persistent production of dozens of proinflammatory molecules. Cytokines, reactive oxygen species (ROS), and many other inflammatory agents produced by adipocytes and immune cells are released and then they activated inflammatory pathways [26]. Hypoxia and oxidative stress are main mechanisms in obesity-induced chronic inflammation [27]. Studies have demonstrated that high-fat diet is a potent inducer of oxidative stress via altering oxygen metabolism. The accumulated intracellular lipid with insufficient oxygen supply stimulates substantial production of ROS and subsequent lipid peroxidation process with toxic metabolites production such as malondialdehyde (MDA) [28]. Antioxidant system was activated at the same time of oxygen stress injury and superoxide dismutase (SOD) is the best known antioxidant enzyme capable of scavenging superoxide radicals, inhabiting cell membrane lipid peroxidation, and neutrophil-mediated inflammation [29]. Another important antioxidant is glutathione peroxidase (GSH-px); it protects the structure and function of cell membrane from peroxide damage by catalyzing glutathione into oxidized glutathione, which makes a poisonous peroxide reduction into nontoxic hydroxyl compounds [30]. In the present study, high-fat diet feeding contributed to the growing body weight and SJP showed prominent effect in protecting against high-fat diet induced obesity. Lipid peroxidation led to increased production of MDA in the heart of obese rats. Although significant increase of MDA was not found in other organs, significant decrease of SOD in the liver, spleen, lung, and kidney of obese rats shed light on the oxidative stress response. From the present point of view, as lipid accumulation continues, macrophages in adipose tissue shift from M2 subtype to a proinflammatory M1 polarization [31], and the macrophages infiltration in adipose tissue was observed at the onset of weight gain [32]. The timely infiltration of macrophages directly contributed to and maintained the inflammatory state of fat, which in turn led to the development of obesity and chronic inflammation [33, 34]. Therefore, the antioxidant system might be initiated at the same time of weight gain and perpetuate throughout the whole process of oxidative response. So, in the process of pro- and anti-inflammation response, the increased metabolites of lipid peroxide in obese rats led to substantial SOD consumption rather than the inflammatory status that inhibited the production of endogenous SOD which might interpret our results. Fortunately, besides losing weight, SJP was efficient in elevating tissue levels of SOD in liver, spleen, and lung. Lowering body weight while improving antioxidant capacity to ameliorate inflammation or attenuating inflammation and then lowering body weight are two aspects that might be in reciprocal causation, just as reported in the papers of Pirola and Ferraz [35] and Hao et al. [24]. However, we did not get expected results in the kidney of obese rats, as there were higher level of GSH-px and lower level of ROS, although the SOD level was still lower than that of rats with normal body weight. The inconsistent results we get in the kidney of obese rats might be due to several factors such as the selection of limited indicator without adequate specificity, or at a certain phase of pro- and anti-inflammation response, or others. However it could not represent the oxidative injuries finally as visualized histologic images displayed clearly more severe damage of kidney with substantial inflammatory cells infiltration and intravascular congestion in obese rats. We think further studies with more specific indicators or longer feeding times will help address this problem.

Obesity-induced inflammatory injuries to organs were unshadowed in the histologic images. We observed distinct changes in different organs of obese rats, such as enlarged hepatocytes, extensive vacuolization, inflammatory cells infiltration, and fatty degeneration in the liver; myocardial edema, early infarction, myocardial cell vacuolization, and granular degeneration in the heart; follicular degeneration and edema in the spleen; edema and bleeding in the lung; substantial inflammatory cells infiltration and fibrogenesis in the kidney and edema; and necrosis and cyst formation in the pancreas. The above changes were attenuated with SJP treatment. In accordance with the present study, numerous studies have demonstrated similar changes in high-fat diet induced obesity and Traditional Chinese herb showed a great potential to improve obesity-induced inflammatory injuries via ameliorating inflammation response [36, 37], modulating microbiota hemostasis [38, 39] and lipid metabolism [40–43], and attenuating insulin resistance [44]. In a part of the above-mentioned Chinese herb formula, we found similar ingredients in SJP, such as rhein [38] and* Curcuma longa* [40], which might be the main effective constituent of the classic formula. However, the specific effective ingredients and mechanisms of SJP in ameliorating obesity-associated inflammatory injuries still need further investigation.

The present study selected MDA, SOD, ROS, MPO, and GSH-px as indicators to reflect the extent of oxidative stress in organs and some changes were detected in selected indicators indeed. More specific indicators of organ damage and serum inflammatory cytokines detection might provide more comprehensive information about obesity-induced systemic inflammation and multiple organ injuries. Although SJP showed an obvious effect in preventing obesity and related multiple organ injuries in the present study, deeper investigation focused on the specific mechanism and effective ingredients basis might enable wide clinical usage.

In conclusion, high-fat diet induced obesity caused extensive inflammatory damage to rats, and SJP was effective in preventing high-fat diet induced obesity and related multiorgan injuries in rats.

## Figures and Tables

**Table 1:** Expression of inflammatory indicators in tissues of rats in the three experimental groups.

Organ	NG (*n* = 6)	OG (*n* = 8)	SG (*n* = 8)
Liver			
MDA (pmol/ml)	1730 ± 258	1755 ± 204	1788 ± 327
ROS (IU/ml)	672 ± 97	707 ± 45	767 ± 56
SOD (U/ml)	321 ± 26	275 ± 9^*∗*^	336 ± 51^#^
GSH-px (mIU/ml)	83 ± 5	68 ± 4^*∗*^	93 ± 4^#^
Heart			
MDA (pmol/ml)	1287 ± 229	1826 ± 76^*∗*^	1512 ± 148^#^
SOD (U/ml)	341 ± 38	363 ± 13	370 ± 12
Spleen			
MDA (pmol/ml)	1403 ± 184	1535 ± 303	1455 ± 254
SOD (U/ml)	271 ± 42	151 ± 24^*∗*^	365 ± 20^#^
Lung			
MDA (pmol/ml)	1549 ± 158	1591 ± 227	1395 ± 166
MPO (ng/ml)	65 ± 9	69 ± 8	68 ± 6
SOD (U/ml)	315 ± 68	208 ± 26^*∗*^	301 ± 46^#^
Kidney			
MDA (pmol/ml)	1437 ± 45	1558 ± 135	1409 ± 215
ROS (IU/ml)	740 ± 164	362 ± 72^*∗*^	552 ± 60^#^
SOD (U/ml)	283 ± 34	229 ± 15^*∗*^	218 ± 35
GSH-px (mIU/ml)	75 ± 19	98 ± 5^*∗*^	72 ± 7^#^
Intestine			
MDA (pmol/ml)	1452 ± 378	1475 ± 262	1591 ± 305
MPO (ng/ml)	68 ± 16	84 ± 4	46 ± 6^#^
SOD (U/ml)	316 ± 38	319 ± 18	237 ± 27^#^
Pancreas			
MDA (pmol/ml)	346 ± 65	321 ± 83	235 ± 48^#^
SOD (U/ml)	167 ± 19	153 ± 29	135 ± 38

MDA: malondialdehyde; SOD: superoxide dismutase; GSH-px: glutathione peroxidase; ROS: reactive oxygen species; MPO: myeloperoxidase. *∗* indicates that, compared with NG, *p* < 0.05; # means that, compared with OG, *p* < 0.05.
